# Modelling Co-Occurring Mental Health Conditions Among Autistic Individuals Using Polygenic Scores

**DOI:** 10.1192/bjo.2024.222

**Published:** 2024-08-01

**Authors:** Adeniran Okewole, Vincent-Raphael Bourque, Sebastien Jacquemont, Varun Warrier, Simon Baron-Cohen

**Affiliations:** 1 Department of Psychiatry, Cambridge University, Cambridge, United Kingdom; 2 Centre Hospitalier Universitaire Sainte-Justine Centre de Recherche, Montreal, Canada; 3 Department of Psychology, Cambridge University, Cambridge, United Kingdom; 4 Autism Research Centre, Cambridge, United Kingdom

## Abstract

**Aims:**

This study investigated the relationship between common genetic variation and co-occurring mental health conditions among autistic individuals.

**Methods:**

The study was conducted with the Simons Foundation Powering Autism Research (SPARK) dataset, V9 release, and included probands [n = 17,582] with confirmed diagnosis of autism, who were also in the SPARK iWES1 array genotyping dataset. Six co-occurring mental health conditions (attention deficit hyperactivity disorder or ADHD, bipolar disorder, depression, schizophrenia, anxiety disorder and disruptive behaviour disorders) were analysed. Polygenic scores (PRS) were generated with PRScs software, using summary statistics from the most recent genome wide association studies (GWAS) for autism, ADHD, schizophrenia, bipolar disorder, depression, anxiety, neuroticism, p-factor, intelligence, educational attainment and hair colour (negative control). General linear models (GLM) and Cox proportional hazards models were computed, with age at registration, sex, cognitive impairment and genetic principal components included in both sets of models. Multiple testing correction was done using the Benjamini-Yekutieli method. Results were calculated using odds ratios (OR), 95% Confidence Intervals (CI) and corrected p values (p).

**Results:**

There were similar patterns of association and interaction for both GLMs and Cox models. Polygenic scores for educational attainment were significantly lower for those with co-occurring ADHD (GLM: OR=8.85E-01, 95% CI=8.48e-01–9.23e-01, p = 2.91E-07; Cox: OR=8.94E-01, 95% CI=8.66e-01–9.22e-01, p = 4.76E-11), bipolar disorder (GLM: OR=7.45E-01, 95% CI=6.54e-01–8.49e-01, p = 2.40E-04; Cox: OR=7.25E-01, 95% CI=6.39e-01–8.23e-01, p = 3.96E-05), depression (GLM: OR=8.63E-01, 95% CI=8.04e-01–9.26e-01, p = 5.13E-04; Cox: OR=8.56E-01, 95% CI=8.03e-01–9.12e-01, p = 2.80E-05), schizophrenia (GLM: OR=6.94E-01, 95% CI=5.71e-01–8.42e-01, p = 3.99E-03; Cox: OR=6.67E-01, 95% CI=5.52e-01–8.05e-01, p = 1.41E-03), anxiety disorder (GLM: OR=8.77E-01, 95% CI=8.37e-01–9.20e-01, p = 9.88E-07; Cox: OR=8.81E-01, 95% CI=8.49e-01–9.15e-01, p = 1.46E-09) and disruptive behaviour disorders (GLM: OR=7.10E-01, 95% CI=6.63e-01–7.60e-01, p = 3.22E-21; Cox: OR=7.10E-01, 95% CI=6.67e-01–7.57e-01, p = 1.35E-24).

**Conclusion:**

Polygenic scores for educational attainment were associated with the co-occurrence of several mental health conditions among autistic individuals.

